# An open-label randomised controlled trial protocol evaluating a Siddha add-on regimen for radiotherapy induced oral mucositis in head and neck cancer patients

**DOI:** 10.3389/fmed.2026.1891612

**Published:** 2026-09-04

**Authors:** Shanmugapriya P, Subathra T, Sowmiya S, Gayatri R, Manish Barvaliya, Sangeetha I, Swathi Subbiah Sankarraj, Ramamurthy M, Kamalasoundaram P, Hariharan S. J, Muthu Prabhakar A, Aleena Babu M, Senthilvel G

**Affiliations:** 1 Department of Nanju Maruthuvam, National Institute of Siddha, Chennai, Tamil Nadu, India; 2 National Institute of Siddha, Chennai, Tamil Nadu, India; 3 Department of Radiation Oncologist, Cancer Institute (WIA) Adyar, Chennai, Tamil Nadu, India; 4 Department of Noi Naadal, National Institute of Siddha, Chennai, Tamil Nadu, India; 5 Department of Health System Research, ICMR-National Institute of Traditional Medicine, Belagavi, Karnataka, India; 6 Department of Integrated Health and Translational Research, National Institute of Siddha, Chennai, Tamil Nadu, India; 7 ICMR-National Institute of Epidemiology, Chennai, Tamil Nadu, India; 8 Cancer Institute (WIA) Adyar, Chennai, Tamil Nadu, India

**Keywords:** integrative oncology, oral mucositis, radiotherapy, RTOG grading scale, Siddha medicine, visual analog scale

## Abstract

Radiation-induced oral mucositis (RIOM) is a common and clinically significant complication in patients undergoing radiotherapy or concurrent chemoradiotherapy for head and neck cancers. It can cause severe oral pain, dysphagia, nutritional compromise, secondary infections, and treatment interruptions, thereby affecting treatment compliance and oncological outcomes. Since the current standard of care (SOC) mainly focuses on symptomatic relief, there is a need for integrative treatment approaches that may improve overall patient outcomes. The present study is designed as a randomised controlled Phase II superiority trial involving 160 participants, with 80 participants allocated to each study arm. Participants in the control arm will receive benzydamine mouthwash six times daily as SOC, while those in the intervention arm will receive Siddha formulations such as *Thiripala Chooranam* mouthwash and *Gungiliya Vennai* topical application three times daily in addition to SOC. Weekly assessments will be carried out by oncologists using validated tools including the Radiation Therapy Oncology Group (RTOG) Oral Mucositis Scale, WHO Oral Mucositis Scale, Visual Analogue Scale (VAS) for oral pain, and WHO three-step analgesic ladder. The primary outcome of the study is the assessment of RIOM severity using RTOG grading scale. Secondary outcomes include evaluation of mucositis severity using the WHO Oral Mucositis Scale, reduction in oral pain assessed by VAS (minimum 2-point reduction), analgesic usage based on the WHO three-step analgesic ladder, safety outcomes, and treatment interruptions. This study may provide evidence on the effectiveness and safety of an integrative Siddha-based approach in reducing RIOM severity and improving supportive care in patients undergoing radiotherapy for head and neck cancers.

**Clinical Trial Registration:**

https://ctri.nic.in/Clinicaltrials/pmaindet2.php?EncHid=MTQ2MTYw&Enc=&userName=, Registered in Clinical Trial Registry, India (CTRI/2025/12/098832- registered on 10-12-2025). Participant enrolment for the trial has commenced, and the study is currently ongoing.

## 1 Introduction

### 1.1 Background and rationale

Head and neck cancer arises from the epithelial lining of the upper aerodigestive tract and represents the seventh most common malignancy worldwide (1). Radiotherapy, either alone or in combination with concurrent chemotherapy, remains a cornerstone in the management of these malignancies (2). However, these treatment strategies are commonly associated with wide range of oral complications, including oral mucositis, secondary infections, xerostomia, dysgeusia, pain and an increased risk of dental and periodontal diseases. These complications impair essential activities like eating, swallowing, speaking and thereby reducing quality of life of patients. In severe cases, they may lead to treatment interruptions, infections and compromised oncological outcomes (3, 4).

Among the complications, oral mucositis (OM) is one of the most dose limiting toxicity of cancer treatment and it is featured by inflammation and ulceration of oral mucosa, typically developing 5–7 days after the initiation of chemo and radiotherapy. Approximately 40% of head and neck cancer patients receiving chemotherapy alone develop OM, whereas 90%–100% patients receiving concurrent chemo-radiotherapy (5). Evidence from Indian clinical settings indicated that radiation-induced oral mucositis (RIOM) usually appears after a cumulative radiation dose of approximately 20Gy, becomes more widespread at around 30Gy, and reaches peak severity with Grade 3 reactions at cumulative doses of 66 – 70Gy (6).

Despite high prevalence and clinical impact of RIOM, the effective curative therapies remain limited. Current management strategies are largely supportive and primarily focus on pain relief and symptomatic management. Conventional interventions include saline mouthwashes, topical anaesthetics, analgesics, cryotherapy, low level laser therapy and protective radiation fields (7, 8). Various pharmacological agents such as benzydamine hydrochloride, doxepin mouthwash and palifermin have been explored for mucositis prevention and treatment. Palifermin, the only US FDA approved drug for OM prevention, is primarily used for hematological malignancies due to high cost and has only 30% reduction in severe mucositis incidence (4, 9). Similarly, benzydamine, chlorhexidine and other anti-inflammatory or anti-microbial washes often provide temporary relief (10). Importantly severe OM frequently results in interruption or modification of cancer therapy, which may adversely affect treatment efficacy (11).

Recent studies have explored the beneficial role of plant-based medicines in the prevention and management of RIOM (12). In this context, the traditional systems of medicine practiced in India may offer promising integrative approaches for RIOM management. The Siddha system of medicine, one of the AYUSH systems traditionally practiced mainly in southern India, as well as in Sri Lanka and Malaysia, includes several therapeutic formulations for the treatment of oral ulcers and inflammatory conditions affecting the oral mucosa. Among these, *Triphala Chooranam* (TPC) mouth wash and *Gungiliya Vennai* (GV) topical application are traditionally indicated for mucosal healing and ulcer management (13, 14). Combining evidence-based Siddha interventions with standard oral care may provide integrative therapeutic approach that enhances mucosal protection, reduces treatment related toxicity and improves compliance in patients undergoing radiotherapy.

TPC, a polyherbal formulation containing *Terminalia chebula* Retz*.*, *Phyllanthus emblica* L*.*, and *Terminalia bellirica* (Gaertn.) Roxb*.*, has supportive evidence suggesting its potential role in the management of oral mucositis. Sruthi et al.*,* conducted a clinical trial among head and neck cancer patients undergoing chemo-radiotherapy demonstrated TPC mouth wash significantly reduced oral pain and associated features when compared to chlorhexidine mouth wash (15). Similarly, Suresh et al.*,* reported a significantly lower incidence of intolerable mucositis among patients receiving TPC mouth wash when compared to povidone iodine (16). GV, another Siddha formulation containing *Shorea robusta* C.F. Gaertn. resin, sesame oil and tender coconut water has demonstrated significant wound healing properties in pre-clinical studies. Bhat et al.*,* reported the topical application of this medicine significantly accelerated regeneration of adnexal skin in animal models indicating strong regeneration and tissue repair that may be beneficial in OM healing (17). Moreover, TPC and *Shorea robusta* C.F. Gaertn. have demonstrated favourable safety profiles in clinical and experimental studies (18–20).

In our previously documented case series (unpublished) involving seven patients, the use of above mentioned Siddha formulations over a period of 48 days, demonstrated notable therapeutic benefits. Summarising the report of that study, patients experienced an average 5-point reduction in pain on Visual Analogue Scale (VAS) and a 2-grade improvement in oral mucositis severity, as measured by WHO Mucositis Grading Scale. These findings suggest the potential value of these Siddha formulations as adjuncts to conventional therapy.

### 1.2 Explanation for the choice of intervention

Considering the high prevalence and clinical burden of RIOM, along with limitations of currently available therapies, there is a need for safe, cost effective and accessible adjunct strategies. Preliminary evidences suggests that TPC and GV possess anti-inflammatory, muco-protective and safety advantages. However, systemic evaluation of their combined use as an adjunct to standard oral care in patients undergoing RT for head and neck remains limited.

Therefore, this study aims to assess the efficacy of a Siddha regimen, comprising TPC mouth wash and GV topical application, as an adjunct to standard of care (SOC), compared with SOC alone, proportionate reduction in the occurrence of severe RIOM, as assessed by the Radiation Therapy Oncology Group (RTOG) grading scale, during radiotherapy and 6 weeks post-radiotherapy, thereby generating scientific evidence to support integrating Siddha practices with SOC.

### 1.3 Objectives

The primary objective of this study is to evaluate the proportionate reduction in the occurrence of severe RIOM, as assessed using RTOG grading scale, among participants receiving a Siddha regimen as an adjunct to standard care compared with those receiving standard care alone, assessed during radiotherapy and at six weeks post-radiotherapy.

The secondary objectives are to assess the proportionate reduction in the severity of radiation-induced oral mucositis using WHO oral mucositis scale at the same time points; to compare oral cavity pain intensity between the study groups using VAS, assessed weekly throughout the study period; to evaluate changes in analgesic usage, including escalation or de-escalation according to the WHO Three-Step Analgesic Ladder, on a weekly basis during the study period; to estimate and compare the proportion of adverse reactions between the study groups; and to evaluate differences in the frequency and duration of treatment interruptions attributable to oral mucositis between the study groups.

## 2 Methods: participants, interventions, and outcomes

### 2.1 Trial setting

The study will be conducted at the Regional Cancer Centre, Adyar, Chennai, India, over a period of 12 months.

### 2.2 Trial design

This study is designed as a superiority, phase II, randomised controlled, open-label, two-arm parallel-group trial comparing participants with RIOM receiving a Siddha regimen as an adjunct to SOC with those receiving SOC alone. The primary endpoint of the study is the reduction in the incidence of severe RIOM, which will be assessed using RTOG grading scale during the course of radiotherapy and again at six weeks after completion of treatment. Participants will be randomly assigned in a 1:1 ratio (Siddha + SOC Vs SOC alone) using variable block sizes to maintain balance between groups, and allocation concealment will be ensured through the sequentially numbered, opaque, sealed envelope (SNOSE) technique. Subgroup analyses are also planned according to treatment modality, including radiotherapy alone, concurrent chemoradiotherapy, and post-operative radiotherapy. Since the study is designed as an open-label trial, centralised assessment of outcomes will be performed wherever possible to reduce the risk of assessment bias.

### 2.3 Eligibility criteria

#### 2.3.1 Inclusion criteria

Participants aged 20–75 years of any gender, diagnosed with head and neck cancers involving the oral cavity and/or oropharynx and scheduled to undergo radiotherapy for a period of 4–7 weeks with a total radiation dose of 55–72 Gy, will be eligible for inclusion in the study. Only those who are willing to participate and are willing to provide written informed consent before the enrolment, will be included.

#### 2.3.2 Exclusion criteria

Participants will be excluded if they have a known hypersensitivity to any ingredient of the Siddha formulation, have active oral infections not related to radiotherapy, or suffer from any severe systemic illness in the view of the investigator may make them unsuitable for participation. Individuals concurrently enroled in another clinical trial, those using dentures, and patients undergoing re-irradiation or palliative radiotherapy will also be excluded from the study.

#### 2.3.3 Criteria for discontinuing or modifying allocated interventions

Participants may be withdrawn from the study prior to completion if they withdraw informed consent at any time without providing a reason, develop serious or intolerable adverse reactions, or experience worsening of RIOM or the emergence of other medical conditions requiring treatment that is incompatible with continued study participation. Participants may also be withdrawn in cases of repeated non-adherence to study procedures, scheduled visits, or the medication regimen, defined as less than 70% drug compliance despite counselling, or if they are unable to be contacted for scheduled assessments despite reasonable efforts.

### 2.4 Interventions and comparator

#### 2.4.1 Intervention description

Arm A will constitute the intervention group, in which participants will receive the Siddha regimen as an adjunct to SOC during the entire course of radiotherapy and the post-radiotherapy period. Participants will be assessed weekly throughout the study duration or until complete recovery from oral mucositis, whichever occurs earlier.

***Procurement of trial drugs:*** The interventional drugs will be procured from GMP certified manufacturers. TPC will be procured from TAMPCOL, Chennai and GV will be procured from IMPCOPS, Chennai.

***Method of drug administration:*** The Siddha intervention will consist of TPC mouthwash and topical application of GV, administered as follows. TPC mouthwash will be prepared using pre-measured teabags, each containing 2 g of TPC. Participants will be instructed to immerse one teabag in 60 mL of freshly boiled water and allow it to stay immersed for 10–15 min, until the solution cools to room temperature. The teabag will then be removed, and the resulting solution will be used immediately as a mouth rinse. Fresh preparation will be performed prior to each use. Then, participants will be instructed to rinse with 60 mL of the mouthwash at each time point, three times daily after meals, swishing for 30seconds and then spitting out the solution. The mouthwash regimen will commence on day0 (one day prior to initiation of radiotherapy) and will continue during radiation therapy and 6weeks post radiation therapy. In addition, GV will be administered at a dose of 10 g per day, applied topically within the oral cavity three times daily, following mouth rinsing. Depending on the clinical schedule, treatment may be initiated on the same day as radiotherapy in some participants.

All participants in Arm A will also receive SOC, which includes 0.15% benzydamine mouth rinse (15 mL for 30–40 s, six times daily), along with topical anaesthetics and anti-inflammatory gels in cases of oral mucositis of grade ≥ 2, as per the institutional protocol. To minimize potential interactions, a standardized administration schedule will be followed. In the intervention arm, 0.15% benzydamine mouth rinse will be used three times daily before meals and three times daily after GV application. TPC mouthwash will be administered at least 30 min after meals, followed by GV application after 10–15 min. Participants will avoid eating or drinking for 30 min after GV application, and adherence will be monitored throughout the study. Details of the investigational drugs and SOC, including dosage specifications, are presented in Table 1.

**Table 1:** List of Siddha interventions and SOC with dose administration details.

S.No	Treatment category	Drug Name	Dose & Vehicle	Total duration
1	Siddha intervention	Thiripala chooranam	180 mL, mouth rinse 3 times per day in divided doses (60 mL per time)	10−13 weeks
2	Siddha intervention	Gungiliya vennai	10gm - 3 times per day in divided doses	10−13 weeks
3	SOC	0.15% benzydamine mouth rinse	15 mL - mouth rinse 6 times per day	10−13 weeks

#### 2.4.2 Comparator description

Participants in Arm B will receive SOC alone for the management of RIOM, as described in the intervention arm. Additional topical anaesthetic and anti-inflammatory gels will be provided for oral mucositis of grade ≥ 2 in accordance with institutional protocol.

Assessments will be conducted weekly, in parallel with the intervention arm, until 6week post radiotherapy or until recovery, whichever occurs earlier. Treatment adherence will be monitored by the oral hygiene coordinator, staff nurse, and investigators, with family caregivers trained during the radiotherapy admission period to assist in monitoring during the post-radiotherapy phase. Trial interventions will be administered for 10–13 weeks, depending on the required radiation dose (Gy) based on tumour size and severity. Recovery will be defined as Grade 0 oral mucositis on both RTOG and WHO oral mucositis scales. All participants will receive radiotherapy using a linear accelerator (LINAC) with three-dimensional conformal radiotherapy (3DCRT) for a duration of 4–7 weeks, in accordance with institutional practice. Before initiation of radiotherapy, all participants will undergo a baseline oral examination and receive necessary dental care, wherever feasible, according to institutional standard dental care protocols. Standardized oral hygiene instructions will be provided and reinforced uniformly throughout the study period in both study arms.

#### 2.4.3 Adherence strategies

Adherence to the intervention and maintenance of oral hygiene will be monitored throughout the treatment period by the staff nurse, oral hygiene coordinator, and study investigators. Family caregivers will be trained during the radiotherapy hospital stay to supervise intervention use and support oral hygiene practices following discharge. Intervention adherence will be documented using a drug compliance form, filled by the investigators during the hospital stay and by caregivers during the 6-week post-radiotherapy follow-up period. During the post-radiotherapy period, compliance will be assessed through telephonic follow-up and by verification of the remaining quantity of dispensed medicine packs at each follow-up visit.

#### 2.4.4 Concomitant care

Concomitant medications considered by the Principal Investigator not to interfere with the efficacy or safety evaluation of the investigational Siddha regimen will be permitted during the study. Participants on stable doses of antihypertensive, antidiabetic, antiepileptic, or cardiac medications may continue their treatment as per standard clinical practice. In the intervention arm, clinically indicated supportive medications, including oral and topical analgesics and anti-inflammatory agents, may be administered to alleviate symptoms and improve patient comfort. All such medications will be prescribed based on the investigator's clinical judgment and in accordance with standard treatment practices. The use of these supportive medications will be appropriately documented.

### 2.5 Outcomes

The primary outcome of the study is the severity of RIOM, assessed using RTOG grading scale. This will be measured as the proportion of participants who develop severe oral mucositis (RTOG grade ≥ 3) at any time during radiotherapy and up to six weeks following completion of radiotherapy, compared between the two study arms. The primary outcome will be assessed from randomisation until the last day of study medication use.

The secondary outcomes include oral mucositis severity assessed using WHO oral mucositis scale, measured as the proportionate reduction in the occurrence and severity of mucositis during radiotherapy and up to six weeks post-radiotherapy. Oral cavity pain, evaluated using VAS as the proportion of participants achieving a minimum two-point reduction in pain intensity and analgesic usage, assessed as the percentage of participants requiring analgesics categorised according to WHO three-step analgesic ladder. Safety outcomes will be evaluated by documenting the number of adverse events reported in each study group. Treatment interruption will be assessed as the proportion of participants experiencing one or more radiotherapy interruptions due to severe RIOM and the total number of days of missed radiotherapy. In addition, body weight changes during radiotherapy, requirement for nasogastric tube feeding, and interruptions in oral intake will be documented as exploratory indicators of nutritional impact. All secondary outcomes, except treatment interruption, will be assessed from randomisation until the last day of study medication use, while treatment interruption will be evaluated until the completion of radiotherapy.

### 2.6 Participant timeline

Participants will undergo initial screening and randomisation at baseline, followed by a 14-week observation period comprising weekly clinical evaluations and periodic laboratory assessments as detailed in the timeline below (Table 2).

**Table 2:** Schedule of enrolment, interventions, and assessments.

Activity/Assessment	Baseline	W1	W2	W3	W4	W5	W6	W7	W8	W9	W10	W11	W12	W13
Enrolment														
Screening & Informed Consent	X													
Randomisation & Allocation	X													
Intervention														
Study Intervention	X	X	X	X	X	X	X	X	X	X	X	X	X	X
Efficacy Assessment														
RTOG Scale	X	X	X	X	X	X	X	X	X					X
WHO oral mucositis Scale	X	X	X	X	X	X	X	X	X	X	X	X	X	X
VAS		X	X	X	X	X	X	X	X	X	X	X	X	X
WHO 3-step analgesic ladder		X	X	X	X	X	X	X	X	X	X	X	X	X
Interruption of RT		X	X	X	X	X	X	X	X					
Days of Missed RT		X	X	X	X	X	X	X	X					
Safety & Laboratory Assessment														
Safety Monitoring		X	X	X	X	X	X	X	X	X	X	X	X	X
CBC	X								X					X
LFT	X								X					X
RFT	X								X					X

RT, radiotherapy; RTOG, Radiation Therapy Oncology Group; WHO, World Health Organization; VAS, visual analogue scale; CBC, complete blood count; LFT, liver function test; RFT, renal function Test; W1−13, No. of week.

### 2.7 Sample size

A study by Kazemiana *et al*. reported that among patients with head and neck cancer receiving benzydamine for the management of RIOM, the occurrence of severe RIOM was 43.6%, compared with 78.6% in the placebo group. Based on findings from our previous unpublished study, a 52% reduction in RIOM grade was observed after 48 days of treatment with TPC mouthwash and GV oral application, and a separate study evaluating TPC with povidone–iodine reported an RIOM occurrence of 10%. Drawing on these findings and clinical expertise, it is assumed that the addition of Siddha regimen to SOC would result in a 15% relative reduction in the occurrence of RIOM compared with SOC alone. Accordingly, assuming a 15% superiority margin, a two-sided significance level of 5%, and 80% power, the calculated sample size is 64 participants per arm. Allowing for an anticipated 20% dropout rate, 80 participants per group will be recruited, resulting in a total sample size of 160 participants. The formula to calculate superiority of proportion is given below.

$$m=\frac{\left(Z\;1\;-\alpha +Z\;1-\beta )\;2\;[\pi S\;(1-\pi S\;)+\pi T\;(1-\pi T\;)\right]}{(\pi T\;-\pi S\;-\delta )\;2}$$

where,

- *δ*: Superiority limit of the difference in Proportions
- π T: Proportion in Test Treatment
- π S: Proportion in Standard Treatment
- π T – π S: Expected difference in proportions
- *α*: Significance level
- 1 – *β*: Power

### 2.8 Recruitment

Following approval of the study proposal and registration with the Clinical Trial Registry of India (CTRI/2025/12/098832- registered on 10-12-2025), investigators are now recruiting participants at the Regional Cancer Centre, Adyar, Chennai.

## 3 Methods: assignment of interventions

### 3.1 Randomisation & sequence generation

Participants will be randomised in a 1:1 allocation ratio to receive either the Siddha regimen with SOC or SOC alone using block randomization with randomly varying block sizes. The randomisation sequence will be generated by an independent statistician using validated online randomisation software. Although randomisation will not be stratified, predefined subgroup analyses based on treatment modality to cancer, will be conducted during outcome analysis to explore potential differential treatment effects across these subgroups.

### 3.2 Allocation concealment mechanism

Allocation concealment will be ensured using SNOSE method. The envelopes will be prepared by an independent statistician not involved in participant recruitment or outcome assessment. All envelopes will be identical in appearance and tamper-proof. The sealed envelopes will be opened one after another only after participant eligibility has been verified and written informed consent has been obtained. Although the trial will be conducted in an open-label method, outcome assessment may be centrally reviewed where feasible to minimise bias.

### 3.3 Implementation

An independent statistician will prepare the randomisation sequence. Participant enrolment and assignment to study groups will be carried out by the study investigator at the recruitment site after confirmation of eligibility and obtaining written informed consent.

### 3.4 Blinding

Considering the nature of the intervention, blinding will not be possible. However, to reduce the risk of assessment bias, outcome evaluation will be carried out by trained assessors who are not involved in treatment allocation or intervention administration and are unaware of the participants' group allocation. Participants will be instructed not to disclose their allocation during assessment visits, and clinical assessments will be completed before application of the topical intervention at each visit. Data analysis will be conducted using coded group identifiers.

## 4 Methods: data collection, management and analysis

### 4.1 Plans for assessment and data collection

Potential participants will be screened by trained investigators to ensure compliance with the inclusion and exclusion criteria. Screening will include a review of medical records and current radiotherapy or chemoradiotherapy details to confirm the diagnosis of head and neck cancer. Eligible participants will be informed about the study objectives, procedures, risks, and benefits, and written informed consent will be obtained prior to enrolment. All screening details will be documented in the screening form and case record form (CRF), with reasons recorded for screen failures. Participants meeting eligibility criteria will proceed to baseline assessments, randomisation, and initiation of the allocated intervention.

At the screening or baseline visit, detailed clinical and drug histories will be recorded. Clinical examination will then be performed to assess the oral mucositis severity using the RTOG and WHO Oral Mucositis Scales during baseline visit. Baseline laboratory investigations, including complete blood count (CBC), liver function test (LFT), and renal function test (RFT), will be performed. Participants will then be randomised, and study medications will be dispensed as per group allocation. Follow-up assessments will be conducted weekly by trained assessors throughout the course of radiotherapy, with telephonic follow-up continued up to six weeks post-radiotherapy. The severity of oral mucositis will be assessed using RTOG and WHO oral mucositis scales, pain intensity will be measured using a 10-point VAS, and analgesic use will be documented according to WHO three-step analgesic ladder. Details of the grading criteria for all these four scales are presented in Table 3. However, in certain clinical situations, analgesics, particularly opioids, may be prescribed for cancer-related pain rather than for RIOM. In such cases, the indication for analgesic use may not directly reflect RIOM severity. To address this, all analgesic usage will be documented along with the primary indication, and cases where analgesics are administered for non-RIOM-related pain, will be clearly specified with appropriate justification. All assessors and physicians involved in outcome evaluation will be trained in advance regarding grading scales and assessment procedures.

**Table 3:** Assessment scales used for outcome evaluation.

Assessment Tool	Grade/Scale	Grading Description
RTOG Oral Mucositis Scale	Grade 0	No change
	Grade 1	Irritation/ Mild pain not requiring analgesics
	Grade 2	Patchy mucositis with inflammatory serosanguinous discharge/ moderate pain requiring analgesics
	Grade 3	Confluent fibrinous mucositis/ severe pain requiring narcotic analgesics
	Grade 4	Ulceration, haemorrhage, or necrosis
WHO Oral Mucositis Scale	Grade 0	No oral mucositis
	Grade 1	Erythema and soreness
	Grade 2	Ulcers present & able to eat solid food
	Grade 3	Ulcers present& requires liquid diet due to mucositis
	Grade 4	Ulcers present; alimentation not possible due to mucositis
VAS for Oral Cavity Pain	0	No pain
	1–3	Mild pain
	4–6	Moderate pain
	7–10	Severe pain
WHO Three-Step Analgesic Ladder	Step 1	Mild pain; Non-opioid analgesics (paracetamol, NSAIDs) ± adjuvants
	Step 2	Mild to moderate pain; Weak opioids (e.g., codeine, tramadol) ± non-opioids and/or adjuvants
	Step 3	Moderate to severe pain; Strong opioids (e.g., morphine, fentanyl, oxycodone) ± non-opioids and/or adjuvants

To evaluate treatment response over time, the percentage distribution of grades/scores obtained from the four above mentioned scales will be assessed separately for each study arm at weekly intervals during study period. These assessments will help compare the severity of mucositis, recovery patterns, pain levels, and analgesic requirements between the two groups over the course of treatment and follow-up. Certain practical limitations may arise while applying the WHO oral mucositis scale in specific clinical situations. For example, some patients may already be receiving nutrition through a nasogastric (NG) tube due to tumour-related obstruction, poor nutritional status, or severe RIOM. In such cases, oral intake is restricted to liquids irrespective of the actual severity of mucositis, which could result in overestimation of the grade, such as categorizing the condition as Grade 3 despite the absence of corresponding clinical findings. To avoid such misclassification, these cases will be recorded as “Not Applicable” for WHO grading, along with proper justification in the study records.

### 4.2 Plans to promote participant retention & complete follow-up

Participant retention will be promoted through regular in-patient engagement during the radiotherapy admission period, enabling close follow-up, supervision of interventions, and minimising participant burden. During the post-radiotherapy period, follow-up will be ensured through scheduled telephonic contacts. Participants and caregivers will receive counselling on the importance of adherence and follow-up, and family caregivers will be trained during radiotherapy to support intervention use after discharge. Reminder calls will be made prior to scheduled visits, and contact details will be verified at enrolment. In the event of missed visits, study staff will attempt prompt telephonic follow-up to collect outcome data wherever feasible, thereby minimising loss to follow-up.

### 4.3 Data management

All study-specific data will be recorded in standardised CRFs. Source documents, including medical records and laboratory reports, will be maintained to allow verification of recorded data. Investigators will document all required participant information in the CRFs, with appropriate justification for any missing data. Data recorded in the CRFs will be routinely cross-verified against source documents, and any discrepancies will be addressed and resolved through data clarification. Treatment details, follow-up information, and scheduled visit dates during post-RT period will be documented in a patient diary. Protocol deviations and the use of concomitant or rescue medications, if any, will also be recorded in the CRFs and reviewed by the investigators at each visit.

Participants who discontinue or deviate from the intervention protocol will continue to be followed, and their data will be included in the final analysis in accordance with the *intention-to-treat* principle. To support participant retention and ensure complete follow-up, regular telephonic communication will be maintained.

#### 4.3.1 Quality assurance

The Principal Investigator will ensure that all data entered in CRFs are accurate, complete, legible, and recorded in a timely manner. To ensure data accuracy, double data entry will be performed for a sub-sample of participants. The data management team will routinely review the database to identify missing values and discrepancies, and CRF entries will be cross-verified against source documents. Participant-specific records will be periodically reviewed to ensure compliance with data recording and documentation standards. Any identified issues will be communicated to the study team, and corrective feedback will be provided to facilitate continuous improvement in data quality.

### 4.4 Plans for collection, laboratory evaluation and storage of biological specimens for genetic or molecular analysis in this trial/future use

No biological specimens will be collected, processed, stored, or used for genetic or molecular analysis as part of this trial or for future research. All laboratory investigations required for clinical assessment, including blood tests, will be performed as part of routine SOC at Regional Cancer Centre and will not involve additional sample collection by the study team.

### 4.5 Statistical methods

Baseline demographic and clinical characteristics of the participants will be summarised using descriptive statistical methods. Categorical data will be presented as frequencies and percentages, whereas continuous data will be expressed either as mean ± standard deviation or as median with interquartile range, based on the distribution of the data.

The primary outcome of the study, namely the severity of oral mucositis assessed using RTOG and WHO oral mucositis scales, will be analysed by comparing the proportion of participants who develop grade ≥2 mucositis in the two study groups. Depending on the suitability of the data, either the chi-square test or Fisher's exact test will be used for comparison. In addition, weekly mucositis grade distributions will be analysed to observe changes in severity, recovery patterns, and overall trends during treatment and follow-up.

For secondary outcomes, pain intensity measured using VAS will be compared between the groups at each assessment point using either the independent samples t-test or the Mann–Whitney U test, based on the data distribution. Analgesic use, categorised according to WHO three-step analgesic ladder, will be analysed as categorical data and compared between groups using the chi-square test or Fisher's exact test. Rescue medication use will be analyzed as a secondary outcome to compare overall analgesic requirements between the study groups. Laboratory investigations will be summarised descriptively and assessed for safety through appropriate within-group and between-group analyses.

The distribution of continuous variables will be checked using histograms to determine normality. Depending on the nature of the data, either parametric or non-parametric statistical methods will be applied for between-group comparisons. Subgroup analyses based on cancer treatment modalities will also be carried out to explore whether the treatment effects remain consistent across clinically relevant subgroups. However, these analyses will be considered exploratory in nature.

Efficacy analysis will follow a modified *intention-to-treat* approach, including all randomised participants who have undergone at least one post-baseline assessment. Safety analysis will be performed using the per-protocol population. A two-sided *p* value of ≤0.05 will be considered statistically significant. All statistical analyses will be carried out by a statistician using the Statistical Package for the Social Sciences (SPSS) software.

## 5 Methods: monitoring

### 5.1 Data monitoring committee

A data monitoring committee (DMC) will be established at the study site, and will function independently of the study investigators. Interim analyses will be conducted during the participant recruitment phase and the same will be submitted to DMC in strict confidence for review and oversight.

### 5.2 Adverse event reporting and harms

Any adverse event occurring after the signing of the informed consent but prior to initiation of the study intervention will be recorded and reported as not related to the study drug. All adverse events occurring from study enrolment until trial completion will be documented and graded using the National Cancer Institute Common Terminology Criteria for Adverse Events (CTCAE), Version 5.0. Any adverse event meeting the criteria for a serious adverse event (SAE) during this period will be reported to the institutional ethics committee (IEC) in accordance with applicable guidelines. Serious adverse events occurring after participant discontinuation will not be reported unless, in the opinion of the investigator, the event is considered possibly related to the study intervention or a study-related procedure. The relationship of adverse events to the study drug will be assessed by the investigators based on temporal association with drug administration and whether the event is unexpected or unexplained in the context of the participant's clinical course, pre-existing medical conditions, and concomitant medications. TPC, when used as an internal medicine, has been associated with suspected adverse drug reactions such as nausea, vomiting, and loose stools, as reported in pharmacovigilance databases. However, no adverse drug reaction reports have been identified for its use as a mouthwash. Based on its safety profile with internal administration, the expected adverse effects in this study may include mild gastrointestinal symptoms such as nausea and vomiting.

#### 5.2.1 Interventions - modifications

Participants who develop adverse reactions that are considered intolerable to the Siddha medicines will have the study intervention discontinued, and the event will be recorded as an adverse reaction. In rare instances of suspected allergic reactions, including skin manifestations, the trial medication will be immediately withdrawn, and the event will be documented as an adverse event. If any biochemical abnormalities are detected during routine laboratory investigations, the study medication will be temporarily withheld until the laboratory values return to normal or clinically acceptable limits. Once the parameters stabilise, the intervention may be restarted at half of the earlier dose, based on the investigator's clinical judgment. In cases where the biochemical abnormalities reappear, the study medication will be permanently discontinued and documented as an adverse event. Even if the assigned intervention is modified or stopped, participants will be encouraged to continue follow-up with the study team so that outcome assessments and relevant study data can still be collected, thereby reducing the possibility of missing data.

### 5.3 Plans for auditing trial conduct

Trial conduct will be monitored periodically to ensure adherence to the approved protocol, Good clinical practice (GCP) guidelines, and ethical requirements. Study records, informed consent, and adverse event reporting will be reviewed for completeness and accuracy. Audits may be performed by the IEC or regulatory authorities, if required. Any deviations identified during monitoring or audit will be documented, and appropriate corrective and preventive actions will be implemented by the investigators.

## 6 Ethics and dissemination

### 6.1 Research ethics approval

Ethical approval for this study has been obtained from the institutional ethics committee (IEC No: IEC/2025/ Nov 01). The protocol, case report forms, and informed consent documents were reviewed and approved for scientific validity and regulatory compliance.

### 6.2 Protocol amendments

Any protocol modifications affecting the conduct of the study, participant's benefits or safety, will require a formal protocol amendment. All such amendments will be submitted to and approved by the institutional ethics committee prior to implementation.

### 6.3 Informed consent

Investigators will explain the study details to eligible patients and provide them with participant information sheets describing the key aspects of the trial. Written informed consent will be obtained from those willing to participate in the study. All participant information sheets and informed consent forms have been prepared in both Tamil and English. In addition, informed oral consent will be obtained from participants prior to enrolment. Participants will be clearly informed that their participation is entirely voluntary, that no incentives will be provided, and that they have the right to refuse participation or decline to answer any questions they find uncomfortable or objectionable, without any penalty or loss of entitled care.

#### 6.3.1 Risk minimization measures

Participants may experience mild discomfort during routine investigations and laboratory tests. Appropriate counselling and support will be provided by the investigators and treating physicians throughout the intervention period. All Siddha medications used in the study will be procured from a GMP-certified pharmaceutical manufacturer.

### 6.4 Confidentiality

All collected information related to the study will be stored confidentially at the study site in locked cabinets with restricted access. To protect participant confidentiality, laboratory samples, reports, case record forms, and other study-related documents will be identified using unique coded numbers rather than personal details. Documents containing identifiable participant information, such as informed consent forms and contact or locator details, will be stored separately from the coded study records. Electronic data will be maintained in password-protected databases with restricted access. In addition, any records that link participant codes with personal identifiers, including logs and appointment registers, will be kept separately in secure locations accessible only to designated personnel.

### 6.5 Ancillary and post-trial care

After completion of RT period, participants in both study arms will be followed for an additional 6weeks, during which the assigned treatments will be continued. Follow-up assessments will be conducted weekly via telephone to monitor recovery and document relevant outcomes.

### 6.6 Availability of data and materials

Study data will be managed by the data management coordinating centre. Principal investigators will have direct access to their respective study datasets. Any additional data access will be governed by institutional policies and applicable ethical and regulatory approvals.

### 6.7 Dissemination plans

The results of the study will be disseminated through peer-reviewed publications and scientific conferences. Dissemination through ICMR-supported academic platforms will be undertaken in accordance with RECAP programme and institutional guidelines, with strict maintenance of participant confidentiality.

## 7 Discussion

RIOM is a common and clinically significant complication in patients undergoing radiotherapy or concurrent chemoradiotherapy for head and neck cancers. It develops due to radiation-induced epithelial injury, inflammatory cytokine activation, and impaired mucosal regeneration, affecting over 90% of patients receiving high-dose treatment, affecting their treatment compliance and oncological outcomes (21, 22). Nevertheless, radiotherapy with or without chemotherapy remains essential for effective locoregional control of head and neck cancers (23). Current SOC for RIOM is largely supportive and mainly focuses on symptom relief. However, these interventions often provide only temporary benefit and may cause adverse effects such as burning sensation and altered taste perception, which can further reduce patient compliance. Therefore, the need for integrative or alternative treatment approaches cannot be ignored (24, 25).

A wide range of medicinal herbs, used either individually or as polyherbal formulations, have been explored for the management of RIOM globally within various traditional medical systems and ethnopharmacological practices (4, 26–28). Systematic reviews report that herbal interventions such as *Glycyrrhiza glabra* L*.*, *Plantago major* L*.*, Hangeshashinto, and *Camellia sinensis* (L.) Kuntze. significantly reduce mucositis severity and pain. Notably, *G. glabra* L. decreased Grade III mucositis by 27.35% compared with controls, while Hangeshashinto reduced incidence by 32% (29). A randomised controlled trial by Saniya C.K. *et al*. (*n* = 60) further demonstrated that *Draksha Guduchyadi Kashaya* significantly delayed the onset of mucositis (*p* = 0.049) and reduced its severity (*p* < 0.001) compared with soda–salt mouthwash, along with improvement in other radiation-induced symptoms (*p* < 0.05). This formulation contains *Vitis vinifera* L., *Tinospora cordifolia* (Willd.) Miers ex Hook. f. & Thomson, *Jasminum grandiflorum* L*.*, and *Berberis aristata* DC*.*, suggesting a synergistic role of polyherbal therapies in mitigating RIOM (30). Additionally, a bio-prospective review of Ayurvedic mouthwash formulations for RIOM identified seven promising plants such as, *Acacia catechu* (L.f.) Willd., *Azadirachta indica* A.Juss*.*, *Glycyrrhiza glabra* L*.*, *Centella asiatica* (L.) Urb*.*, *Emblica officinalis* Gaertn., *Terminalia belllirica* (Gaertn.) Roxb*.*, and *Terminalia chebula* Retz., supporting further therapeutic investigation (31).

Across these studies, a consistent observation is that effective herbal interventions predominantly possess astringent, bitter, and sweet tastes, which correlate with anti-inflammatory, antimicrobial, and wound-healing properties beneficial in RIOM. From a Siddha perspective, high-energy radiation acts as a severe external etiological factor that disrupts *aiyam* (water-earth humour responsible for lubrication and mucosal protection), particularly the mucosal protective component analogous to *bodhaga aiyam* (a sub-type of *aiyam*), leading to *udal thathu azhivu* (degeneration or destruction of body tissues) characterised by epithelial desquamation and basement membrane damage. The resulting structural depletion aggravates *vali* (manifesting as dryness, ulceration, pain) and *azhal* (manifesting as inflammation, burning, erythema), culminating in RIOM (32). The present study is designed to evaluate a Siddha regimen, TPC mouthwash and GV topical oral application, as an adjunct to SOC in the management of RIOM. This integrative approach is grounded in Siddha principles, as the formulations predominantly exhibit bitter and astringent tastes and is further supported by emerging biomedical evidence demonstrating the anti-inflammatory, antioxidant, antimicrobial, and wound-healing properties, with prior clinical and preclinical data indicating their relevance in mucosal or dermal injury (33–35).

Previous pre-clinical studies do indicate that TPC is useful as a radioprotective agent and can protect mice from radiation-induced sickness and mortality (36, 37), intestinal mucosal damage (38) as well as reduce radiation-induced DNA damage in both mice and cultured mammalian cells (39, 40). Earlier clinical studies evaluating TPC alone or in combination with other polyherbal formulations in the management of RIOM have demonstrated promising therapeutic effectiveness. A cumulative analysis of two studies evaluated Oro-T oral rinse, containing TPC as the main ingredient, in patients undergoing radiotherapy/chemotherapy. More than 80% of patients reported symptomatic improvement and 95% showed investigator-assessed clinical improvement. In contrast, the normal saline group showed worsening symptoms in 92.31% (patient-reported) and 84.62% (investigator-assessed) cases. Oro-T delayed onset, reduced RIOM severity, and improved quality of life without adverse effects. However, small sample size (40 vs. 15), pooled study designs, and restricted randomisation limit generalizability (41).

A comparative study of 36 Head & Neck Squamous Cell Carcinoma patients undergoing chemo-radiotherapy evaluated TPC mouthwash (*n* = 18) against 2% chlorhexidine mouthwash (*n* = 18) for 21 days, using WHO oral mucositis scale and patient reported outcome measure scale (PROMS) for assessment. Patients using TPC showed greater improvement in quality of life and reduction of oral side effects, particularly mucositis, compared with chlorhexidine, suggesting comparable therapeutic effectiveness and supporting its use as an adjunct therapy in the present study (15).

Similarly, GV is traditionally indicated for ulcers and inflammatory lesions. An experimental study in Sprague Dawley rats (*n* = 32) with excision wounds (300 mm²) evaluated the wound-healing effects of GV and *Kalchunna Thailam* (KT) compared with mupirocin and untreated controls. Both GV and KT significantly hastened epithelialisation (GV: 15.83 days; KT: 15.17 days vs. control: 21.33 days; *p* < 0.001), with GV additionally demonstrating enhanced vascularity and regeneration of adnexal skin structures such as hair follicles, sweat glands, and sebaceous glands, indicating potent wound-healing potential (17). Also, GV is indicated for burn wounds in Siddha literatures (13). RIOM shares key pathological features with burn injuries, including oxidative stress, inflammatory cytokine activation, epithelial damage, and delayed re-epithelialisation, suggesting that agents with proven wound-healing activity in burn models may also be beneficial in RIOM.

Importantly, the safety profile of both TPC and *Shorea robusta* C.F. Gaertn*.,* based preparations is well established. Toxicological evaluations of TPC in Sprague Dawley rat models, including acute, and long-term studies up to 270 days, have demonstrated no significant toxic effects, suggesting a favourable safety profile and potential suitability for long-term use (18). At the oral dose of 2,500 mg/d, TPC had no serious adverse effects in healthy volunteers (42). Moreover, Acute toxicity study on *Shorea robusta* C.F. Gaertn*.* shows that LD50 was more than 2000mg/kg in Sprague-Dawley rats (19). Another toxicity study conducted in mice and Wistar rats demonstrated that the formulation is safe for human consumption, based on 14- and 21-day toxicity evaluations showing no significant adverse effects (20).

The present study is designed as a randomised controlled Phase II superiority trial. One of its major strengths is the use of multiple validated tools for comprehensive assessment of RIOM. RTOG and WHO oral mucositis grading scales provide physician-based evaluation of mucosal severity and functional impairment, while the VAS captures patient-reported pain. In addition, WHO analgesic ladder reflects the need for analgesic support related to RIOM pain. Together, these measures allow a multidimensional assessment of disease severity and progression. Weekly assessments during radiotherapy and follow-up will help to closely monitor changes in mucositis severity and recovery over time. The study also evaluates treatment interruptions, an important but often under-reported consequence of severe RIOM that may affect overall treatment outcomes.

Certain limitations of the study should also be considered. Since the trial follows an open-label design, there is a possibility of performance and reporting bias. However, the use of standardised assessment scales, blinded outcome assessment and evaluation by trained oncology professionals are expected to minimise this risk. In addition, published evidence on interventions comparable to the present Siddha regimen for RIOM is limited. Although preliminary institutional observations were referenced to provide contextual understanding, they were not used as formal evidence or assumptions for sample size estimation. The study may also encounter certain clinical and assessment-related challenges during the study period. Some participants may require NG tube feeding of liquid food due to conditions such as tumour obstruction, nutritional compromise, postoperative status, or severe RIOM, which may lead to overestimation of severity grading, particularly with WHO oral mucositis scale. Similarly, additional supportive medications administered according to institutional protocols and cancer-related complications may affect symptom severity and outcome assessment. These factors will be carefully documented and considered during analysis.

## 8 Conclusion

In conclusion, this study is an effort to scientifically evaluate the role of Siddha medicine as an adjunct to contemporary oncology care in the management of RIOM. The study aims to address an important supportive-care need through the use of accessible, affordable, and culturally acceptable interventions. If Siddha regimen is found to be more effective than SOC alone in reducing severe RIOM, pain, and treatment interruptions without causing additional toxicity, it may contribute to improving patient-centred care in head and neck cancer radiotherapy settings. The findings may also help support the development of integrative oncology practices and future national guidelines.
